# Class-specific interactions between Sis1 J-domain protein and Hsp70 chaperone potentiate disaggregation of misfolded proteins

**DOI:** 10.1073/pnas.2108163118

**Published:** 2021-12-03

**Authors:** Hubert Wyszkowski, Anna Janta, Wiktoria Sztangierska, Igor Obuchowski, Tomasz Chamera, Agnieszka Kłosowska, Krzysztof Liberek

**Affiliations:** ^a^Intercollegiate Faculty of Biotechnology of University of Gdansk and Medical University of Gdansk, University of Gdansk, Gdansk 80-307, Poland

**Keywords:** Hsp40, protein aggregation, chaperones

## Abstract

Protein homeostasis is constantly being challenged with protein misfolding that leads to aggregation. Hsp70 is one of the versatile chaperones that interact with misfolded proteins and actively support their folding. Multifunctional Hsp70s are harnessed to specific roles by J-domain proteins (JDPs, also known as Hsp40s). Interaction with the J-domain of these cochaperones stimulates ATP hydrolysis in Hsp70, which stabilizes substrate binding. In eukaryotes, two classes of JDPs, Class A and Class B, engage Hsp70 in the reactivation of aggregated proteins. In most species, excluding metazoans, protein recovery also relies on an Hsp100 disaggregase. Although intensely studied, many mechanistic details of how the two JDP classes regulate protein disaggregation are still unknown. Here, we explore functional differences between the yeast Class A (Ydj1) and Class B (Sis1) JDPs at the individual stages of protein disaggregation. With real-time biochemical tools, we show that Ydj1 alone is superior to Sis1 in aggregate binding, yet it is Sis1 that recruits more Ssa1 molecules to the substrate. This advantage of Sis1 depends on its ability to bind to the EEVD motif of Hsp70, a quality specific to most of Class B JDPs. This second interaction also conditions the Hsp70-induced aggregate modification that boosts its subsequent dissolution by the Hsp104 disaggregase. Our results suggest that the Sis1-mediated chaperone assembly at the aggregate surface potentiates the entropic pulling, driven polypeptide disentanglement, while Ydj1 binding favors the refolding of the solubilized proteins. Such subspecialization of the JDPs across protein reactivation improves the robustness and efficiency of the disaggregation machinery.

## Significance

How chaperones rescue cells from toxic aggregates, associated with stress, aging, and disease, is not fully understood. Here, we focus on aggregate recognition by yeast Hsp70- and Hsp104-disaggregating proteins. We show that two conserved classes of J-domain proteins (JDPs/Hsp40s), which regulate Hsp70, use disparate mechanisms to recruit chaperones to aggregates. Bipartite interaction with Hsp70 enables Sis1, a Class B JDP, to tether more Hsp70 molecules to the aggregate, which improves disaggregation with Hsp104. Ydj1 of the Class A, in turn, drives effective reactivation of misfolding-prone substrates. Our results demonstrate that the two classes of JDPs, albeit overlapping in function, differently contribute to the individual stages of disaggregation. This demonstrates how the diversification of cochaperones improves protein quality control.

Molecular chaperones are involved in the maintenance of protein homeostasis by aiding correct protein folding (1). Yet severe stress conditions induce excessive protein misfolding and aggregation (2). Upon stress relief, the return to the proteostasis is mediated by the Hsp70 chaperone with cochaperones, including J-domain proteins (JDPs/Hsp40s), which together restore the native state of misfolded polypeptides trapped in aggregates (3–5). The JDP–Hsp70 system acts alone in metazoans or in cooperation with an Hsp100 disaggregase in most other eukaryotes and bacteria (5, 6).

Protein disaggregation and refolding starts with a recognition of misfolded polypeptides within an aggregate by a JDP, and then, its J-domain interacts with the nucleotide-binding domain of Hsp70, inducing ATP hydrolysis which triggers the closure of the Hsp70’s substrate-binding domain over the aggregated substrate (7, 8). The aggregate-bound Hsp70 interacts with an Hsp100 disaggregase, and this interaction allosterically activates Hsp100 and tethers it to the aggregate (9–16). Subsequently, in an ATP-driven process, Hsp100 disentangles and translocates polypeptides from aggregates (17–21), which enables their correct refolding, spontaneous or with an assistance of Hsp70 and its cochaperones (22, 23).

JDPs are the major regulators of the Hsp70 activity and substrate specificity (3, 24, 25). In yeast *Saccharomyces cerevisiae*, a general Hsp70 chaperone, Ssa1, is recruited to protein disaggregation by two main cytosolic JDPs, Ydj1 and Sis1, assigned to the Class A and Class B, respectively (3, 4, 26). Both Ydj1 and Sis1 comprise a helical, highly conserved J-domain, a flexible, mostly unstructured G/F region, two beta-barrel peptide-binding domains, CTDI and CTDII, and a C-terminal dimerization domain (27–33). Ydj1 additionally features a Zn-binding domain located in the first part of the CTDI region of the protein, which is distinctive for the Class A JDPs (32, 34).

Despite the structural similarities, the two JDPs are functionally nonredundant. Sis1 is essential, and Ydj1 is required for growth above 34 °C (26, 27, 35, 36). Overexpression of Sis1 suppresses the phenotype caused by the deletion of *YDJ1*, while Ydj1 overexpression is not sufficient to suppress the deletion of *SIS1* (26, 27, 35–37). The two JDPs show different specificities toward amorphous and amyloid aggregates (35, 38) and different populations of amorphous aggregates formed in vitro (4, 24).

Recent reports shed more light on the JDPs’ divergence. Both JDPs form homodimers, which differ in the structural orientation of the J-domain: In Sis1, the J-domain is restrained from Hsp70 binding by the interaction with the Helix 5 in the G/F region (26, 33, 39–41). Such autoinhibition, which also occurs in most human Class B JDPs, is released through the interaction with the C-terminal EEVD motif of Hsp70 (33, 42). This regulation is important for the disassembly of amyloid fibrils by the human JDP–Hsp70 system (43), but its role in the handling of stress-related, amorphous aggregates is not clear. Despite the breadth of data on Hsp70 mechanisms, we still lack understanding of how the disparate features of the JDPs impact Hsp70 functioning in protein disaggregation.

Here, we investigate individual steps of protein disaggregation in the context of functional differences between Sis1 and Ydj1. Using various biochemical approaches, we show that the two JDPs drive different modes of Ssa1 binding to aggregated substrates, which dictate diverse kinetics of their disaggregation by Hsp104. The distinctive performance of Sis1 is associated with its interaction with the C terminus of Hsp70. Our results suggest that the bivalent interaction with the Class B JDP conditions aggregate remodeling by the Hsp70 system, resulting in enhanced Hsp104-dependent protein recovery. Our data indicate a mechanism by which the Class A and B JDPs contribute to the disaggregation efficacy in a complex and divergent manner.

## Results

### Sis1 and Ydj1 Exhibit Distinct Influence on Disaggregation and Aggregate Binding.

**Fig. 1.**
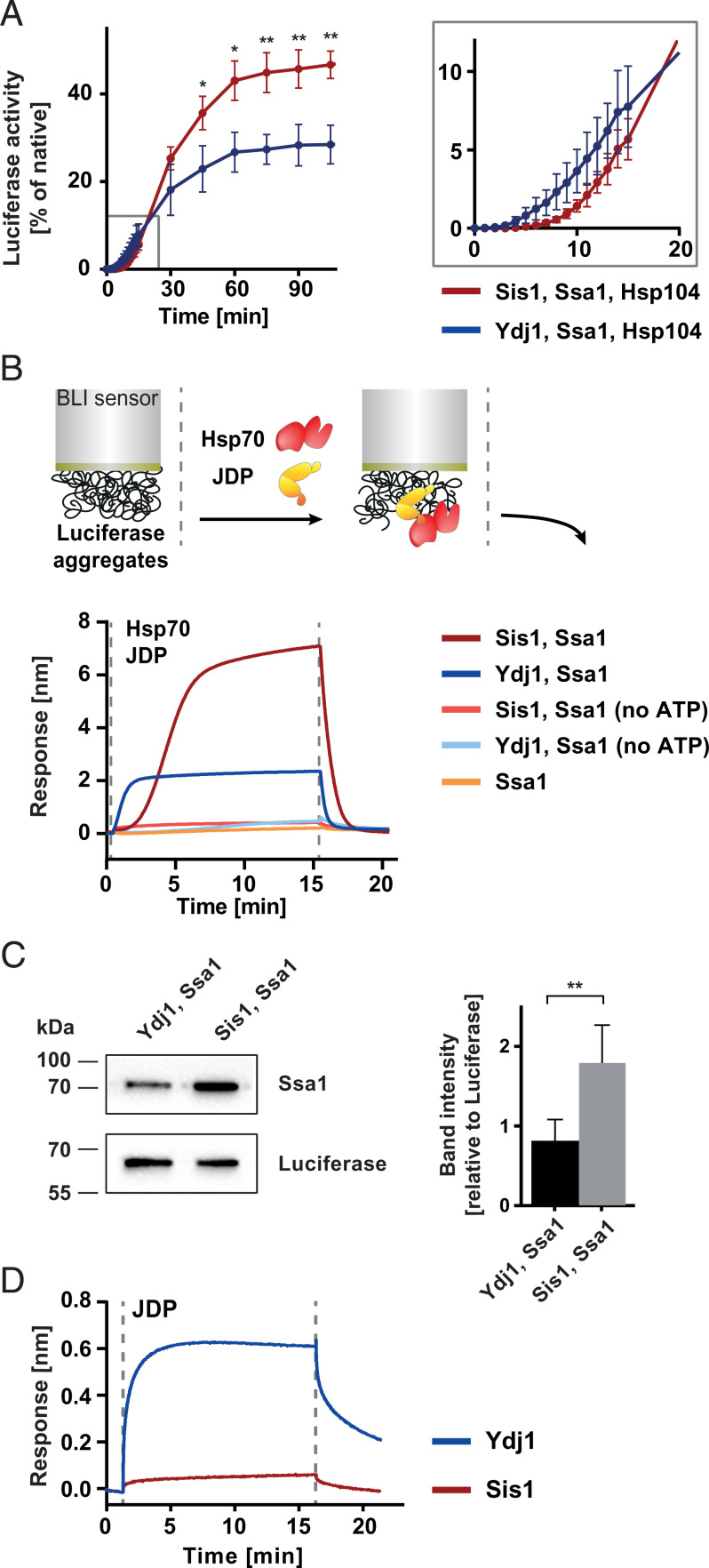
Hsp70 system with Sis1 or Ydj1 shows differences in aggregate binding and disaggregation. (*A*) Refolding of aggregated luciferase by Ssa1 and Hsp104 in the presence of Sis1 (red) or Ydj1 (blue). (*Inset*) Magnification of the first 20 min of the reaction. Error bars show SD from three experiments. Luciferase activity was normalized to the native protein activity. Two-tailed *t* test: **P ≤ 0.01 and *P ≤ 0.05. (*B, Upper*) The scheme of the experiment. BLI sensor with immobilized luciferase aggregate was incubated with Ssa1 and Sis1 or Ydj1, with or without ATP, as indicated in the legend. Dashed lines indicate the start of the incubation with the chaperones and washing with the buffer without chaperones. Shown is a representative result for three repeats. (*C*) Western blot analysis of Ssa1 binding in the presence of Sis1 or Ydj1 to the sensor with luciferase aggregates. The experiment was performed as in *B*, except that at the end of the binding step the proteins dissociated from the sensor into the Laemmli buffer and were analyzed with Western blot. (*Right*) The bands intensities were quantified. Error bars show SD from three independent experiments. Two-tailed *t* test was performed: **P ≤ 0.01. (*D*) Binding of Sis1 (red) and Ydj1 (blue) without Ssa1 to luciferase aggregates immobilized on the BLI sensor, performed analogously as in *B*.

To understand the differences between the Sis1 and Ydj1 functioning in protein disaggregation, we monitored the recovery of aggregated protein substrates, luciferase and GFP, by the Hsp104–Ssa1 chaperone system with either of the JDPs. The experiment revealed kinetic differences in protein reactivation: While the reaction with Ydj1 proceeded at a high rate shortly after the addition of the chaperones, protein recovery with Sis1 followed a few-minutes-long lag phase (Fig. 1*A* and *SI Appendix*, Fig. 1*A*). Furthermore, the disaggregation machinery with Sis1 eventually yielded a higher level of reactivated protein (Fig. 1*A* and *SI Appendix*, Fig. 1*A*). The effect was observed across a wide range of JDP concentrations (*SI Appendix*, Fig. 1 *B* and *C*). We observed a similar trend when we measured the protein-refolding activity of Sis1–Ssa1 and Ydj1–Ssa1 in the absence of Hsp104 (*SI Appendix*, Fig. 1*D*). With Sis1, the recovered luciferase activity reached a four times higher level than with Ydj1, and the curve had a sigmoidal shape with ∼7 min of lag.

To gain more insight into the functional differences between the JDPs at the initial step of substrate processing, we employed biolayer interferometry (BLI). This technique analyzes changes of thickness of a protein layer at the surface of an optical sensor and, therefore, allows to observe the time-resolved binding of chaperones to a protein that had been immobilized and heat-aggregated on the sensor (44). The Sis1–Ssa1 system showed delayed binding to aggregated luciferase, reaching the thickness of ∼7 nm (Fig. 1*B*). Such effect was not observed for Ydj1–Ssa1, whose binding was rapid but reached only one-third of the level of Sis1–Ssa1 (Fig. 1*B*). A similar, chaperone-binding pattern was observed across a wide range of JDP and Ssa1 concentrations (*SI Appendix*, Fig. 1 *E* and *F*), as well as for other protein substrates: GFP aggregates and heat-aggregated proteins from yeast cell lysate (*SI Appendix*, Fig. 1 *G* and *H*). ATP was required for both systems, which is consistent with the ATP dependence of Hsp70 (Fig. 1*B*). Consistently, the Ssa1 mutant deficient in ATP hydrolysis and substrate binding, Ssa1 T201A V435F (45, 46), was not recruited to the aggregate by the JDPs (*SI Appendix*, Fig. 1*I*).

The higher binding signal in the presence of Sis1 suggests more Ssa1 molecules bound to the aggregate than with Ydj1, which was confirmed with the Western blot analysis of the sensor-bound Ssa1 (Fig. 1*C*). These results indicate that Ydj1 and Sis1 impose different modes of Ssa1 recruitment to the aggregated substrate.

### Aggregate Modification by Hsp70 with Class B JDP Improves Chaperone Binding to Aggregate.

The classical model of the Hsp70 substrate-binding cycle involves initial recognition and binding of a protein substrate by a JDP, which then promotes its binding to Hsp70 (47). To understand the molecular events that cause the differential JDP behavior, firstly, we studied the ability of the JDPs to bind to the aggregated substrate in the absence of Ssa1. Surprisingly, only Ydj1 showed evident binding to luciferase aggregates (Fig. 1*D*), while the Sis1–aggregate interaction was detected at a very low level using, in parallel, the Sis1 F201H mutant with impaired substrate binding as a reference (*SI Appendix*, Fig. 1*J*) (48).

**Fig. 2.**
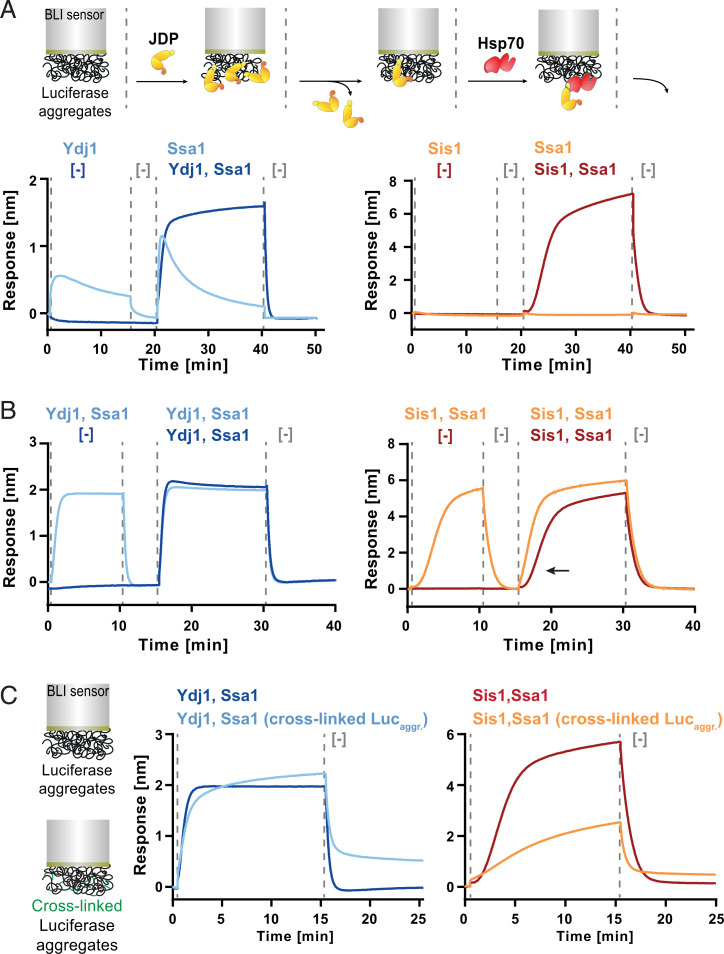
Sis1 and Ydj1 drive different modes of Ssa1 recruitment to aggregates. (*A, Upper*) The experimental scheme. BLI sensor with luciferase aggregates was initially incubated with or without Ydj1 (*Left*) or Sis1 (*Right*). After washing, the sensor was incubated with Ssa1 or JDP-Ssa1, as indicated in the legend. (*B*) Sequential incubation of luciferase aggregates, immobilized on the sensor with the indicated chaperones, was carried out analogously as in *A*. The effect of the initial incubation on the binding kinetics of Sis1-Ssa1 is indicated with an arrow. (*C*) Sensors with luciferase aggregates after glutaraldehyde cross-linking, and untreated were incubated with Ydj1-Ssa1 or Sis1-Ssa1. Dashed lines indicate the starting point of each step. The presented results are representative for three replicates.

To investigate whether the JDPs bound to the substrate are effective in the Hsp70 recruitment, next, we incubated Sis1 or Ydj1 with the sensor containing luciferase aggregates, followed by a washing step and subsequent incubation with Ssa1 without JDPs. The aggregate-bound Ydj1 promoted binding of Ssa1, whereas there was no Hsp70 binding when the aggregate had been initially incubated with Sis1 (Fig. 2*A*). Overall, this shows that Ydj1 binds aggregates in a more stable manner and rapidly recruits Hsp70 to the substrate, while in the case of Sis1, the interaction with an aggregated substrate requires the JDP and Hsp70 to be present simultaneously.

We hypothesized that this obligatory collaboration in aggregate binding by Sis1–Ssa1 and the long lag phase might reflect the following features of the system: 1) slow association rate between Sis1 and Ssa1 in the solution limits their binding to the aggregate and 2) Sis1–Ssa1 binding to the aggregate promotes the cooperative recruitment of more chaperones, either through multimeric chaperone–chaperone interactions or through aggregate modifications that gradually expose more sites compatible for chaperone binding or both. To test the first possibility, we incubated Sis1–Ssa1 together for 30 min prior to the addition to the sensor-bound aggregate, which proved to have no effect on the binding (*SI Appendix*, Fig. 2*A*). Furthermore, when we immobilized Ssa1 directly on the BLI sensor, Sis1 bound to it rapidly (*SI Appendix*, Fig. 2 *B* and *C*). Thus, the rate of the Sis1–Ssa1 complex formation likely does not limit its binding to the aggregate.

Next, to assess whether it might be aggregate remodeling that determines the binding pattern of the Hsp70 system, we carried out two subsequent incubations of the sensor-bound aggregate with Sis1–Ssa1, reasoning that the chaperones would bind more rapidly to a surface that had already been processed by this Hsp70 system. When we incubated the sensor-bound aggregates with Sis1–Ssa1 and, after chaperone dissociation, we introduced Sis1–Ssa1 again, we observed binding without the lag (Fig. 2*B*). We performed an analogous experiment with Ydj1–Ssa1, but the first incubation did not influence the second (Fig. 2*B*).

To further verify whether the improved substrate binding by Sis1–Ssa1 is associated with a modification of aggregate structure, we tested whether restraining aggregated polypeptides mobility with cross-linking would exert any effects specific to Sis1–Ssa1. Indeed, glutaraldehyde cross-linking of the sensor-bound aggregate significantly reduced Sis1–Ssa1 binding, while only a minor change was observed for Ydj1–Ssa1 (Fig. 2*C*).

Next, we analyzed if the size, mass, or shape of aggregates is distinctly affected by the JDPs. After an incubation of luciferase aggregates with Ssa1 and the JDPs, we subjected them to sedimentation in a glycerol gradient. Sis1–Ssa1 caused a small fraction of aggregated luciferase to remain at the top of the gradient, similarly, as does native luciferase, while it also yielded a population of protein species that was observed across the gradient (*SI Appendix*, Fig. 2*D*). In case of Ydj1–Ssa1, no luciferase was detected on the top and only trace amounts in the middle of the gradient.

These results strongly suggest that Sis1–Ssa1 initially changes aggregate structure in a way that favors cooperative and more efficient binding of the Hsp70 system.

### Initial Processing by Hsp70 with Class B JDP Makes Aggregate-Trapped Polypeptides More Amenable to Disaggregation.

**Fig. 3.**
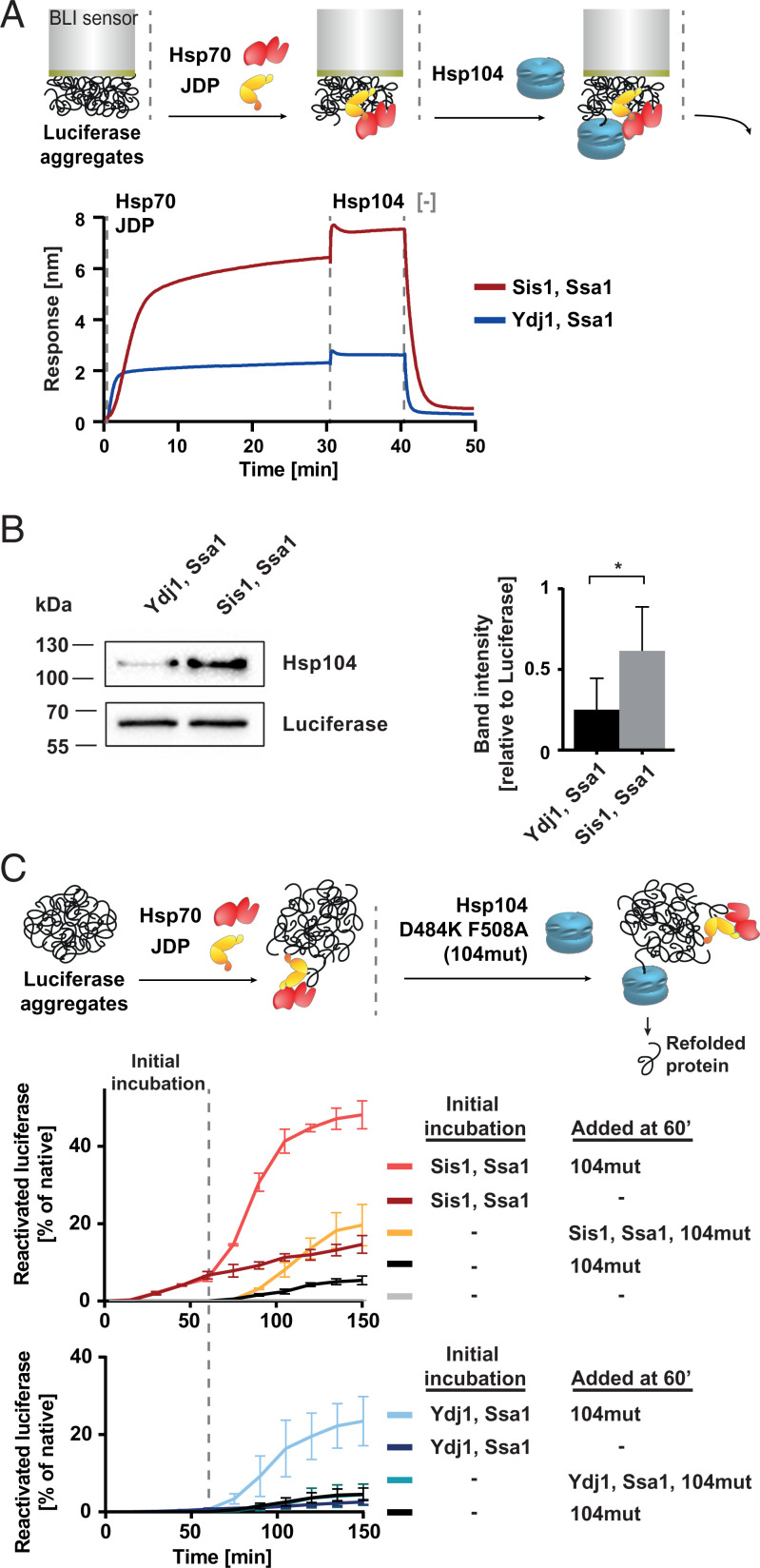
Sis1 facilitates the initiation of Hsp104-dependent protein disaggregation. (*A*) BLI sensor covered with luciferase aggregates, and Sis1-Ssa1 (red) or Ydj1-Ssa1 (blue) was incubated with Hsp104. Washing step involved the buffer without chaperones. The presented results are representative for three replicates. (*B*) Western blot analysis of Hsp104 binding in the presence of Ssa1 and Sis1 or Ydj1 to the sensor covered with luciferase aggregates. The experiment was performed as in *A*, except instead of the dissociation step the proteins were removed from the sensor by boiling the sensor in Laemmli buffer, and subsequently, the Western blot was performed using anti-Hsp104 and anti-luciferase (control) antibodies. (*Right*) The bands intensities were quantified. Error bars show SD from three independent experiments. Two-tailed *t* test was performed: *P ≤ 0. 05. (*C*) Impact of initial incubation with the Hsp70 system on the efficacy of disaggregation of denatured luciferase. Luciferase activity was measured at the indicated time points. 104mut designates the Hsp104 D484K F508A variant. Luciferase activity was normalized to the native protein. Error bars show SD from three independent experiments. (*Upper*) Experimental schemes. Dashed lines indicate the starting points of the incubation steps.

In previous studies, it has been shown that the efficacy of disaggregation depends strongly on how effectively Hsp104 binds to the substrate, which in turn largely relies on the Hsp104 interaction with Hsp70 (9, 44, 49). Knowing that Sis1–Ssa1 remodels aggregates in a way that results in more Hsp70 bound to the aggregate surface (Fig. 1 *B* and *C*), we asked whether it promotes binding of proportionally more Hsp104 molecules. To test this, the BLI sensor with luciferase aggregates was incubated with the JDP–Hsp70 system and, subsequently, Hsp104 was added. The Hsp104-binding signal was about three times higher in case of Sis1–Ssa1 than for Ydj1–Ssa1, which was observed across various of Hsp104 concentrations (Fig. 3*A* and *SI Appendix*, Fig. 3*A*). By measuring the amount of Hsp104 on the sensor with Western blot, we confirmed that more Hsp104 is bound to the aggregate with Sis1–Ssa1 (Fig. 3*B*). This suggests that the aggregate-remodeling activity of Sis1–Ssa1 not only promotes Ssa1 association with aggregated substrates but also leads to the more efficient recruitment of Hsp104, which ultimately improves protein disaggregation. Agreeably, when aggregates were cross-linked with glutaraldehyde and their remodeling by Sis1–Ssa1 or Ydj1–Ssa1 was minimized, Hsp104 binding to the Hsp70-covered aggregates was also strongly reduced (*SI Appendix*, Fig. 3*B*).

Next, we wanted to assess whether the Hsp70-dependent remodeling of protein aggregates affects disaggregation in any other way than through chaperone accumulation on the substrate. To address this, we used the Hsp104 D484K F508A variant of the disaggregase with disrupted Hsp70 binding, which does not require this interaction to be allosterically activated (44). As shown with BLI, Hsp104 D484K F508A is capable of binding to the aggregate independently of Hsp70, albeit weakly, and the presence of the JDP-Hsp70 system does not increase but slightly inhibits its binding (*SI Appendix*, Fig. 3*C*). Theoretically, the JDP–Hsp70 system should not stimulate disaggregation by this Hsp104 variant—unless the aggregate surface is modified in a way that makes the substrate more manageable by the disaggregase. To investigate that, we initially incubated luciferase aggregates with JDP–Hsp70, and then, we added the mutant Hsp104. Such preprocessing by Hsp70 with either Sis1 or Ydj1 for 1 h enhanced luciferase disaggregation by Hsp104 D484K F508A (Fig. 3*C*). Strong stimulation was also observed with aggregated GFP (*SI Appendix*, Fig. 3 *D*–*F*), which is curious in the light of the fact that the JDP–Hsp70 system alone does not yield any detectable recovery of GFP fluorescence. This reveals an unapparent manifestation of JDP–Hsp70 chaperone activity—one that does not produce a refolded protein but changes an aggregate into a better substrate for the Hsp104 disaggregase variant.

In the above experiments, aggregate preprocessing by Sis1–Ssa1 had a superior impact on the efficacy of protein reactivation in comparison with Ydj1–Ssa1 (Fig. 3*C* and *SI Appendix*, Fig. 3 *D*–*F*). Even when the Sis1–Ssa1 system was added to the aggregates simultaneously with Hsp104 D484K F508A, after the characteristic lag, positive effect was observed, suggesting that Hsp70 can remodel aggregates in parallel with an ongoing disaggregation by Hsp104. To additionally verify that the stimulation results from the remodeling of aggregates and not from the Hsp104 activation by Ssa1 nor facilitated polypeptide refolding downstream of the disaggregase, we modified the assay to inhibit Hsp70 at the moment of the addition of the Hsp104 D484K F508A to the reaction. Ssa1 activity strongly depends on potassium ions (50), and therefore, it is sensitive to sodium at the level that only moderately affects Hsp104. When the Hsp70 activity was minimized with 120 mM sodium chloride, Hsp70 slightly inhibited the disaggregase, and the stimulation of disaggregation was observed only when the aggregates had been preprocessed by Hsp70 in the buffer without NaCl (*SI Appendix*, Fig. 3*G*).

These results show that the initial aggregate remodeling by JDP–Hsp70, in particular with Sis1, significantly contributes to the refolding capacity of the disaggregation system.

### Interaction between Sis1 CTDI and Ssa1 EEVD Is Crucial for Aggregate Remodeling.

**Fig. 4.**
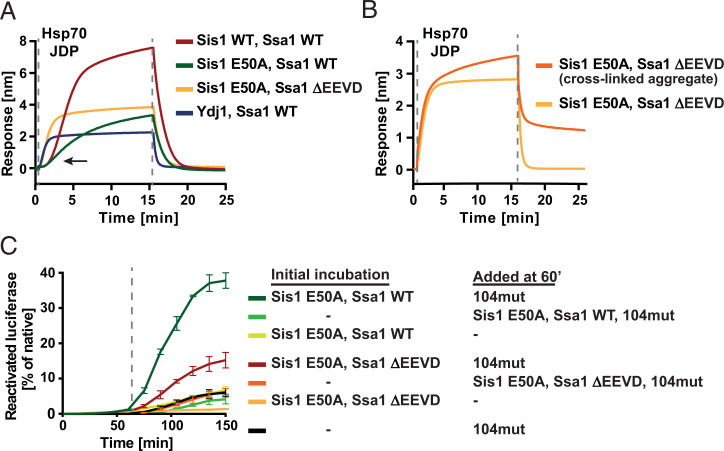
Sis1 (CTDI)-Ssa1 (EEVD) interaction is crucial for the superior, aggregate-remodeling activity of Sis1-Ssa1. (*A*) Binding of chaperone variants (as color indicated in the legend) to luciferase aggregates immobilized on the BLI sensor, according to the scheme in Fig. 1*B*. (*B*) Binding of Sis1 E50A and Ssa1 ΔEEVD to luciferase aggregates cross-linked with glutaraldehyde (orange) and nonmodified (yellow), as on the scheme in Fig. 2*C*. The presented results are representative for three replicates. (*C*) Luciferase reactivation by the system upon sequential addition of different chaperone variants (as color indicated in the legend), according to the scheme in Fig. 3*C*. Luciferase activity was normalized to the native protein. Error bars show SD from three independent repeats. Dashed lines indicate the starting points of the subsequent steps of experiments.

Next, we set out to establish what are the molecular basis of the observed discrepancies between Ydj1 and Sis1. Both of the JDPs interact with the ATPase domain of Hsp70 through the HPD motif in the J-domain (30, 51). It has also been shown before that the HPD motif is critical for cooperation with Hsp70 (47, 51, 52), and not surprisingly, when we substituted HPD with AAA in Sis1, Ssa1 recruitment to the aggregate was minimal (*SI Appendix*, Fig. 4*A*).

However, contrary to Ydj1, Sis1 additionally interacts with Ssa1 through the CTDI domain, which recognizes the C-terminal EEVD motif of Ssa1 (31, 33, 42, 53). Deletion of the EEVD motif abrogates the interchaperone collaboration, hampering Hsp70 binding both through Sis1 CTDI and the J-domain, as the latter is restrained by the interaction with the Helix 5 of the G/F region (31, 33, 42). Agreeably, we observed that the ΔEEVD mutation is detrimental for Sis1–Ssa1 binding to the aggregate but does not have any effect on the Ydj1–Ssa1 system (*SI Appendix*, Fig. 4*B*). Furthermore, when Sis1 was directly immobilized on the sensor, it interacted with Ssa1 wild type (WT) but not with Ssa1 ΔEEVD (*SI Appendix*, Fig. 4*C*). Consistent results were obtained with a reversed setup (*SI Appendix*, Fig. 4*D*).

We asked whether the Ssa1 EEVD interaction site in Sis1 governs the distinctive behavior of this JDP in aggregate binding and remodeling by the Hsp70 system. To restore the Ssa1 activation through the J-domain, despite the EEVD deletion, we introduced in Sis1 a mutation that would destabilize the Helix 5 and unlock the J-domain analogously, as has recently been done for its human ortholog, DNAJB1 (*SI Appendix*, Fig. 4*E*) (33). However, while DNAJB1 ΔH5 fully restored the luciferase disaggregation activity with Hsp70 ΔEEVD (33), Sis1 ΔH5–Ssa1 ΔEEVD neither bound to nor recovered aggregated luciferase (*SI Appendix*, Fig. 4 *F* and *G*). These results, and the fact that the G/F regions in Sis1 and DNAJB1 are not entirely conserved (*SI Appendix*, Fig. 4*E*), suggest that the regulation of the J-domain inhibition might be differently tuned in the two orthologs.

Another Sis1 variant, E50A, has been previously described to recover luciferase disaggregation with Ssa1 ΔEEVD because of the disrupted J-domain inhibition by the G/F region (42). The disruption of the autoinhibition was at least partial, as indicated by the later NMR study of the analogous variant of DNAJB1 (33). Accordingly, the E50A mutation increased the level of Ssa1 ΔEEVD binding, albeit it was still much lower than with Ssa1 WT (*SI Appendix*, Fig. 4*H*). To verify if the improved interaction with Ssa1 ΔEEVD resulted from Sis1 E50A being better recognized as a JDP, rather than as a destabilized protein substrate, we assessed the Sis1 interaction with the Ssa1 T201A V435F variant deficient in ATP hydrolysis and substrate binding. Compared with Sis1 WT, Sis1 E50A binding to Ssa1 T201A V435F was slightly increased (*SI Appendix*, Fig. 4*H*), suggesting that the E50A mutation does not make Sis1 a better protein substrate for Ssa1, which would be manifested in the reduced binding to Ssa1 T201A V435F. This supports the more available J-domain as the primary cause of the more efficient Ssa1 ΔEEVD binding.

The E50A mutation in Sis1 also restored the Ssa1 ΔEEVD binding to the sensor-bound aggregate, although Sis1 E50A was unable to sustain as efficient chaperone docking as the WT system (Fig. 4*A*), which points to the relevance of the J-domain regulation in this regard. Interestingly, the aggregate-binding curve of Sis1 E50A–Ssa1 ΔEEVD closely resembled that of Ydj1–Ssa1 WT and Ydj1–Ssa1 ΔEEVD (Fig. 4*A* and *SI Appendix*, Fig. 4*B*). At the same time, Sis1 E50A–Ssa1 WT binding to the aggregate retained the long lag characteristic for the WT Sis1 (Fig. 4*A*), indicating that the autoinhibition mechanism is not critical for this feature. This suggests that the Sis1 (CTDI)–Ssa1 (EEVD) interaction itself determines the sigmoidal kinetics of the Hsp70 recruitment to the aggregate.

While the structural confinement of the aggregate with cross-linking had adverse effects on the WT Sis1–Ssa1 binding (Fig. 2*C*), it only slightly affected Sis1 E50A–Ssa1 ΔEEVD (Fig. 4*B*), similarly as Ydj1–Ssa1 (Fig. 2*C*). This suggests that once the Sis1 (CTDI)–Ssa1 (EEVD) interaction is disrupted, aggregate modification does not either take place or play a significant role in chaperone binding. We also examined the relevance of this interaction for aggregate remodeling by Hsp70 that aids Hsp104-dependent disaggregation. While the initial incubation of luciferase or GFP aggregates with Ssa1 WT and either the WT or E50A variant of Sis1 strongly improved disaggregation by Hsp104 D484K F508A, the stimulation by Sis1 E50A–Ssa1 ΔEEVD was much weaker (Fig. 4*C* and *SI Appendix*, Fig. 4*I*), lower than observed for Ydj1–Ssa1 (Fig. 3*C* and *SI Appendix*, Fig. 3*F*).

Together, these results imply that the chaperone activity of Sis1–Ssa1, superior to Ydj1–Ssa1 in the aggregate binding and remodeling prior to the recruitment of the disaggregase, largely depends on the accessory interaction between the CTDI of the JDP and the C terminus of Hsp70.

## Discussion

Results presented in this work show that JDPs of two classes, Ydj1 (Class A) and Sis1 (Class B), dictate the course of the Hsp70 system activity with protein aggregates. While the systems with either of the JDPs are functional in protein refolding (4, 29), they display disparate mechanisms of interaction with aggregated substrates. Ydj1 follows the classical Hsp70 cycle, in which a substrate is first bound by the JDP and then loaded onto Hsp70. With Sis1, which does not bind aggregates efficiently on its own, the formation of the chaperone complex at the aggregate is delayed but yields loading of far more Hsp70 and Hsp104 molecules. The distinctions of Sis1 strictly depend on the interaction between the CTDI domain of the JDP and the EEVD motif in Ssa1.

**Fig. 5.**
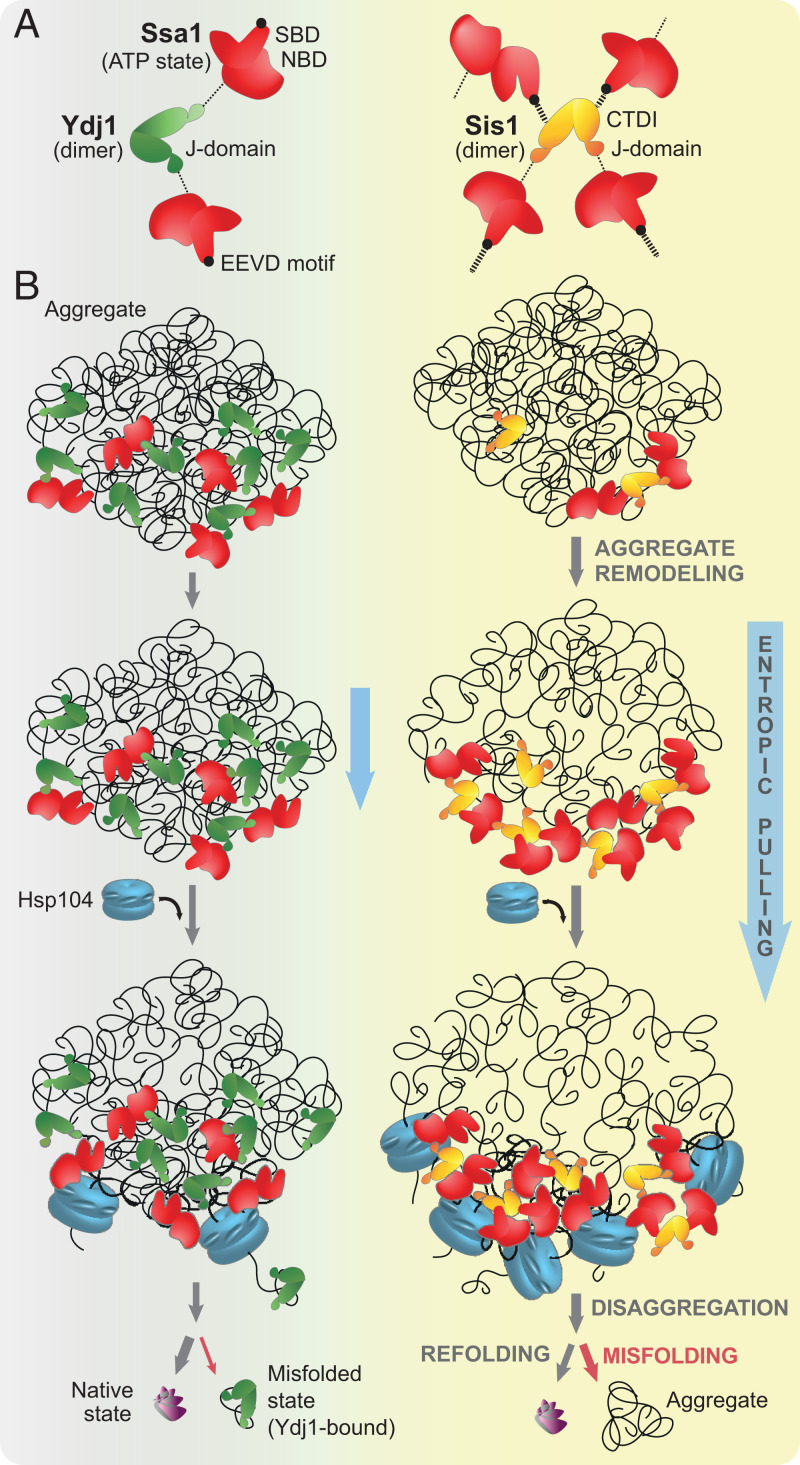
Model of distinctive contribution of Class A and B JDPs to protein disaggregation. (*A*) While both Ydj1 and Sis1 bind and stimulate Hsp70 through the J-domain binding to Ssa1 NBD, in Sis1, the additional interaction with the EEVD motif expands the network of possible interactions. (*B*) Despite the initially a lower level of aggregate binding, with time, Sis1 enables more abundant loading of Ssa1 molecules on the aggregated substrate. Larger chaperone complexes likely potentiate entropic pulling (54), which remodels aggregates in a way that favors more abundant chaperone binding. Saturation of protein aggregate with Hsp70 enhances Hsp104 docking and disaggregation. Disaggregated polypeptides with misfolding proclivity are better substrates for Ydj1 than Sis1 (26). Ydj1 binding prevents their aggregation, which promotes their return on correct folding pathways.

How could this accessory Sis1–Ssa1 interaction lead to the binding of more chaperones to aggregates? Theoretically, the Ydj1 dimer could bind only two Hsp70 molecules at once with its two J-domains. In Sis1, the accessory Ssa1 binding site offers the chance for an expanded interaction network that could form larger chaperone complexes at the aggregate surface (Fig. 5*A*). In favor of that, the Sis1 (CTDI)–Ssa1 (EEVD) interaction appears to be much stronger than the canonical one through the J-domain (*SI Appendix*, Figs. 2*B* and 4 *C* and *D*), and since it is not sensitive to ADP nor lack of nucleotides, it is likely unaffected by the nucleotide state of Hsp70. In comparison with Ydj1–Ssa1, Sis1–Ssa1 interacted strongly (*SI Appendix*, Figs. 2 *B* and *C* and 4*H*), although it is not clear whether the two sites in Sis1 are structurally fit to interact with the same Hsp70 molecule. If they are, the interaction with EEVD could increase the effective local concentration of the J-domains, which would explain the efficient Ssa1 binding to the aggregate, even at lower-Sis1 concentrations (*SI Appendix*, Fig. 2 *C*, *E*, and *F*). Such Sis1–Ssa1 coupling could also compensate for the very weak Sis1–aggregate interaction (Fig. 1*D* and *SI Appendix*, Fig. 1*J*) by favoring the activation of substrate binding domain (SBD) closure over a polypeptide initially recognized by Ssa1, not the JDP. All these Sis1 properties may build up the power to assemble more Ssa1 on the aggregate.

The abundance of Hsp70 molecules on the aggregate may have at least two positive effects on the efficacy of substrate refolding (Fig. 5*B*). Firstly, more Hsp70 at the aggregate supports avid binding through multiple subunits of hexameric Hsp104 (9–11, 13–16, 44). Agreeably, we observed the improved Hsp104 binding to the Sis1–Ssa1-covered aggregates (Fig. 3 *A* and *B* and *SI Appendix*, Fig. 3*A*). Secondly, Hsp70 crowding on aggregates may potentiate entropic pulling of the chaperone-bound polypeptides (24, 54). Such effect has been recently shown for Hsp70 and Hsp110 chaperones that were densely clustered on amyloid fibrils, which augmented fibril disassembly (43). Accordingly, our results indicate that the assembly of larger complexes of Ssa1 with Sis1 lead to what we refer to as aggregate remodeling. It might involve partial or complete disentanglement of polypeptides from aggregates, although the molecular nature of such aggregate modification is not clear. Some insight may come from a recent study (55), which demonstrates that, in a cell, Sis1 overexpression recruits Ssa1 to the aggregates of heat-denatured luciferase, as well as the inclusions of polyglutamine tracts, and allows the chaperone to penetrate to the core of these assemblies in a liquid-like manner, suggesting that their structure becomes less densely packed. The herein observed changes in aggregate sedimentation upon JDP–Hsp70 treatment suggest similar effect (*SI Appendix*, Fig. 2*D*). We also functionally probed the JDP–Hsp70-dependent aggregate remodeling using the Hsp104 variant that associates with aggregates and solubilizes substrates autonomously from Hsp70. Initial incubation with Sis1–Ssa1 significantly enhanced the refolding capability of this variant, suggesting that the misfolded polypeptide chains might become more exposed to Hsp104 or easier to extract from aggregates or both. The Ydj1–Ssa1 system also had a positive effect on disaggregation, but the stimulation was weaker, possibly because the chaperone complexes with Ydj1 are smaller, and the associated entropic pulling has a reduced aggregate-remodeling potential (Fig. 5*B*).

Aggregate modification might also explain why aggregate binding by Sis1–Ssa1 accelerates with time: The substrate-bound Hsp70 exerts force that exposes additional, chaperone-binding sites. This scenario is supported by the lack of efficient Sis1–Ssa1 binding to the aggregates in which disentanglement of polypeptides has been limited with cross-linking (Fig. 2*C*).

Furthermore, our results obtained with the Sis1 E50A–Ssa1 ΔEEVD variants indicate that the remodeling is impaired upon the disruption of the Sis1 (CTDI)–Ssa1 (EEVD) interaction, which is manifested in the lack of the delay in aggregate binding, minor sensitivity to aggregate cross-linking, and weaker stimulation of the Hsp104 D484K F508A mutant (Fig. 4 *A*–*C* and *SI Appendix*, Fig. 4*I*). In all these aspects, the mutant Sis1 E50A–Ssa1 ΔEEVD system is similar to Ydj1–Ssa1, in which the JDP collaborates with Ssa1 only through the J-domain. Sis1 is different as it operates through two-step binding, with the autoinhibition of the J-domain being released upon Ssa1 binding through the EEVD motif. Recently, a detailed work on the human Sis1 homolog, DNAJB1, showed that its regulation through the autoinhibition is important for the superior disaggregation of amyloid fibrils with human Class B JDPs (33). Does disruption of this regulation also affect reactivation of stress-aggregated proteins? Although, in DNAJB1, the E50A substitution restored the chaperone activity with the Hsp70 ΔEEVD variant, only the ΔH5 mutation fully released the J-domain (33). However, because the G/F region is not well conserved, it is difficult to estimate the degree of the J-domain release in the Sis1 variants based on DNAJB1. Unlike in DNAJB1, the disruption of the Helix 5 in Sis1 diminished the luciferase disaggregation activity with both Ssa1 WT and Ssa1 ΔEEVD (*SI Appendix*, Fig. 4*G*). This suggests that either the autoinhibition is critical for any Sis1–Ssa1 activity or that the generated, long, unstructured region, which comprises 18 residues more than in DNAJB1 ΔH5, interferes with Sis1 folding or stability. The former is unlikely, given that Sis1 E50A cooperates with Ssa1 ΔEEVD, which is a clear sign of impaired autoinhibition, and yet it remains as active as the WT Sis1 in the luciferase disaggregation with Ssa1 WT (42). At the same time, we observed that the E50A mutation reduces aggregate binding by Sis1–Ssa1 WT (Fig. 4*A*), while it does not have a major impact on aggregate remodeling (Figs. 3*C* and 4*C*). Thus, the autoinhibition mechanism might play a role in the Hsp70 recruitment to the aggregate surface, while the advantage of Sis1 in the latter process seems to rely predominantly on its interaction through CTDI with the EEVD motif of Ssa1.

Nonetheless, it is the J-domain, with the key HPD motif, that is central to the JDP function–Hsp70 activation. Interestingly, although the mutation of HPD in Sis1 hampers the chaperone activity (30) and aggregate binding (*SI Appendix*, Fig. 4*A*), it has been recently shown that it does not fully abrogate the ability to interact with polyglutamine aggregates in the cell (55). It suggests that the complex of Sis1 with Ssa1 (EEVD), even without the Hsp70 activation, might confer a function, at least with some protein substrates, especially in the context of other JDPs that may simultaneously cochaperone the same Hsp70. Such collaboration through Hsp70 might contribute to the synergy that has been observed for the Hsp70 system with mixed JDPs, associated with the interclass JDP–JDP interactions (4).

Although Sis1 similarly improves chaperone binding to the luciferase and GFP aggregates (Fig. 1*B* and *SI Appendix*, Fig. 1*G*), it is curious why the disaggregation of GFP was much more efficacious with Sis1 than with Ydj1 (*SI Appendix*, Fig. 1*A*), while the advantage of Sis1 in the reactivation of aggregated luciferase was not as strong (Fig. 1*A*). These discrepancies might come from different substrate requirements for the Hsp70 assistance in refolding: While GFP folds spontaneously without chaperones, luciferase does not (29, 56). We propose that the JDPs distinctively shape folding pathways of polypeptides after they have been processed by the disaggregase. Ydj1 on its own is an efficient “holdase”: It binds to unfolded substrates, stabilizes their folding intermediates, and thus prevents their misfolding and aggregation (29). Agreeably, Ydj1-bound aggregates rapidly and stably (Figs. 1*D* and 2*A*), which might confer advantage under acute stress. Sis1 has also been reported to bind misfolded substrates alone, albeit with low affinity (26, 29), and we observed only marginal interaction with the aggregate (Fig. 1*D*). Moreover, in contrast to Ydj1, Sis1 requires Ssa1 for aggregation inhibition (4, 29, 33). In line with that, the system with Ydj1 is substantially more effective than Sis1 in the refolding of nonaggregated, misfolded luciferase, and this trend is conserved across the evolution of Class A and B JDPs (4, 24, 29). Our results explain why this trend is reversed in the recovery of refolding-competent proteins, such as GFP, when protein reactivation is limited by disaggregation, at which Sis1 is much more effective.

In summary, distinct activities that are prompted by Sis1 and Ydj1 might be significant at different stages of protein recovery from aggregates. Through an expanded network of interactions, Sis1 can more efficiently harness Hsp70 to predispose protein aggregate for processing by the disaggregase by means of aggregate remodeling and decorating it with Hsp104-binding sites (Fig. 5 *A* and *B*). Upon polypeptide disaggregation and release from Hsp104, reaggregation of problematic folding intermediates can be prevented by Ydj1. This model illustrates how protein aggregation, a hallmark of deterioration of protein homeostasis, can be more successfully neutralized in eukaryotes thanks to the expansion and divergence of JDP cochaperones.

## Materials and Methods

### Proteins.

Published protocols were used to purify Hsp104 (57), Ssa1 (58), Sis1 (59) Ydj1 (57), His-tagged luciferase (44), and GFP (60). Point mutations were introduced using PCR site-specific mutagenesis (Qiagen) and confirmed with sequencing. All the described protein concentrations refer to monomer. Creatine kinase was purchased from Sigma-Aldrich (10127566001). Untagged luciferase was purchased from Promega (E1701).

### Luciferase Refolding Assay.

Luciferase (1.875 mg/mL) was chemically denatured in the buffer A (25 mM Hepes-KOH pH 8.0 75 mM KCl, 15 mM MgCl_2_) with 6-M Urea and incubated at 25 °C for 15 min. Subsequently, it was transferred to 48 °C, incubated for 10 min, and rapidly diluted 25 times with the buffer A. The reactivation reaction was initiated by adding aggregated luciferase (0.2 µM, final concentration) to the reaction mixture containing chaperones. All chaperones were used at 1 µM concentration (unless specified differently), except Hsp104 D484K F508A, which was used at 0.5 µM. Luciferase activity was measured using Luciferase Assay Kit (E1501, Promega) with Sirius Luminometer (Berthold). The curves presented in the figures represent averages with SD from at least three experiments. Statistical analysis was done using the GraphPrism software.

### Western Blotting.

Chaperone binding to the aggregate-covered sensor was examined with Western blot as follows: Just before the dissociation step in the BLI experiment, the sensor was removed from the reaction buffer and incubated for 10 min at 100 °C in the Laemmli buffer (4% SDS, 20% glycerol, 10% 2-mercaptoethanol, 0.004% bromophenol blue, and 0.125 M Tris HCl, pH 6.8) with 50 mM EDTA. Polyacrylamide electrophoresis in denaturing conditions (SDS-PAGE) and immunoblotting were carried out according to the standard procedures. Rabbit anti-sera specific to Hsp104 and Ssa1 were used as primary antibodies. Anti-rabbit IgG conjugated with horseradish peroxidase (HRP) (Bio-Rad) were used as secondary antibodies. Blots were developed using SuperSignal West Pico Chemiluminescent Substrate (Thermo Fisher Scientific), scanned using ChemiDoc MP Imaging System (Bio-Rad), and quantified using Image-Lab software (Bio-Rad). Statistical analysis was done using the GraphPrism software.

### BLI.

Aggregate-binding experiments were performed as previously described (44), using the BLItz and Octet K2 instruments (ForteBio). The Ni-NTA biosensor (ForteBio Dip and Read) was initially hydrated with the buffer A; next, it was immersed for 10 min in the buffer A with 6-M urea and 8.2 µM His-tagged luciferase. Within this time, the binding of luciferase reached saturation at the biolayer thickness of ∼6 nm. After washing with the buffer A for 5 min, the biosensor was transferred to the buffer A containing 1.6 µM native His-tagged luciferase and incubated at 44 °C for 10 min, which resulted in a luciferase aggregate of the thickness of ∼16 nm. The biosensor was then equilibrated for 10 min with the buffer A containing 2 mM DTT and 5 mM ATP.

To prepare biosensors with cross-linked aggregates, the sensor with aggregated luciferase, after washing with the buffer A for 5 min, was incubated in the buffer A with 0.1% glutaraldehyde for 5 min and washed in the buffer A for 5 min again.

The baseline, chaperone binding, and dissociation steps were performed in the buffer A with 2 mM DTT and 5 mM ATP, unless indicated differently. Chaperones were used at 1 µM concentration (unless specified differently). Saturating JDPs and Ssa1 concentrations of 1 µM (*SI Appendix*, Fig. 1 *E* and *F*) were chosen to measure the maximum binding capacity of the chaperone system for the same amount of aggregated substrate.

The background chaperone binding to the BLI sensors was additionally tested using His_6_-SUMO, to which neither JDPs nor Ssa1 bound, based on the BLI experiment (*SI Appendix*, Fig. 5 *A* and *B*, *Left*). To assess the chaperone binding to the sensor outside the biolayer surface (e.g., glass walls), we incubated the sensor with immobilized His_6_-SUMO in the buffer A with chaperones. The sensor was subsequently analyzed with Western blot. Association of Ssa1 and both JDPs was detected (*SI Appendix*, Fig. 5 *A* and *B*, *Right*). The level of Ssa1 with or without JDPs was similar, unlike the binding to the sensor with immobilized aggregates, in the case of which Ssa1 binding in the presence of Sis1 was significantly higher (Fig. 1*C*). This suggests that the residual JDPs associated with the sensor outside the aggregated substrate did not recruit Ssa1 (*SI Appendix*, Fig. 5*B*).

## Supplementary Material

Supplementary File

## Data Availability

All study data are included in the article and/or *SI Appendix*.
