# Supplementary material for: Class-specific interactions between Sis1 J-domain protein and Hsp70 chaperone potentiate disaggregation of misfolded proteins
Source: Proc Natl Acad Sci U S A. 2021 Dec 3;118(49):e2108163118. doi: 10.1073/pnas.2108163118 (PMC8670446; doi:10.1073/pnas.2108163118)
Supplement: Supplementary File [file pnas.2108163118.sapp.pdf]

Supplementary Information

Class-Specific Interactions Between Sis1 J-Domain Protein and Hsp70 Chaperone Potentiate Disaggregation of Misfolded Proteins

Hubert Wyszkowski, Anna Janta, Wiktoria Sztangierska, Igor Obuchowski, Tomasz Chamera, Agnieszka Kłosowska*, Krzysztof Liberek*

Supplementary Figures

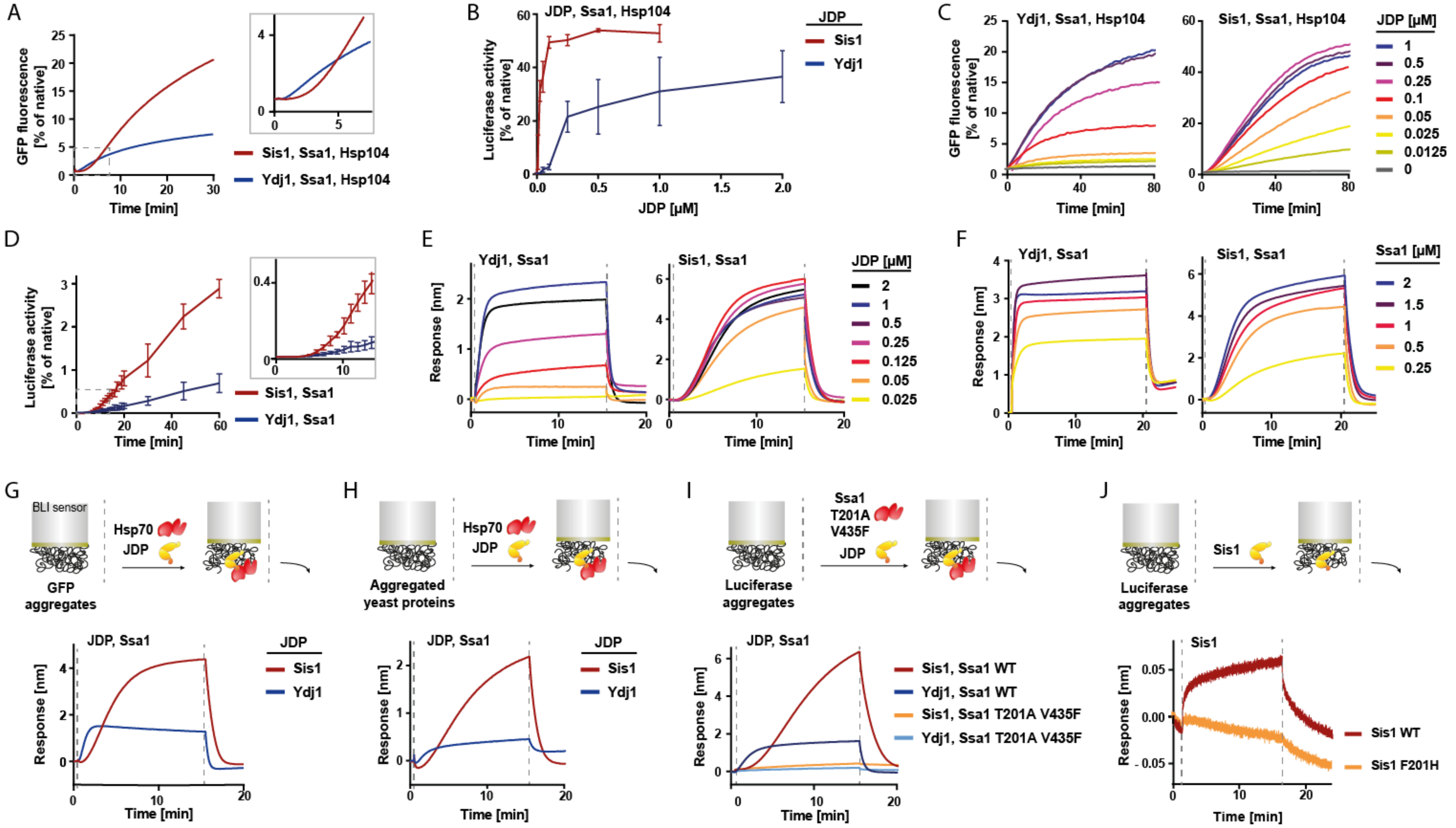
A
GFP fluorescence [% of native]
Time [min]
Sis1, Ssa1, Hsp104
Ydj1, Ssa1, Hsp104
B
JDP, Ssa1, Hsp104
Luciferase activity [% of native]
JDP [µM]
JDP
Sis1
Ydj1
C
Ydj1, Ssa1, Hsp104
Sis1, Ssa1, Hsp104
GFP fluorescence [% of native]
Time [min]
JDP [µM]
1
0.5
0.25
0.1
0.05
0.025
0.0125
0
D
Luciferase activity [% of native]
Time [min]
Sis1, Ssa1
Ydj1, Ssa1
E
Ydj1, Ssa1
Sis1, Ssa1
Response [nm]
Time [min]
JDP [µM]
2
1
0.5
0.25
0.125
0.05
0.025
F
Ydj1, Ssa1
Sis1, Ssa1
Response [nm]
Time [min]
Ssa1 [µM]
2
1.5
1
0.5
0.25
G
BLI sensor
Hsp70
JDP
GFP aggregates
JDP, Ssa1
Response [nm]
Time [min]
JDP
Sis1
Ydj1
H
Hsp70
JDP
Aggregated yeast proteins
JDP, Ssa1
Response [nm]
Time [min]
JDP
Sis1
Ydj1
I
Ssa1 T201A V435F
JDP
Luciferase aggregates
JDP, Ssa1
Response [nm]
Time [min]
Sis1, Ssa1 WT
Ydj1, Ssa1 WT
Sis1, Ssa1 T201A V435F
Ydj1, Ssa1 T201A V435F
J
Sis1
Luciferase aggregates
Response [nm]
Time [min]
Sis1 WT
Sis1 F201H

Supplementary Figure 1. (A) Renaturation of heat-aggregated GFP in the presence of Hsp104, Ssa1 and Ydj1 or Sis1. Inlet shows the enlarged initiation of the reaction. (B) Reactivation of aggregated luciferase by Ssa1 and Hsp104 at the increasing JDPs concentrations. Error bars show SD from three experiments. (C) Disaggregation of heat-aggregated GFP by Ssa1, Hsp104 and the JDPs at the indicated concentrations. (D) Reactivation of aggregated luciferase by the Hsp70 system without Hsp104. Inlet shows the enlarged initiation of the reaction. Error bars show SD from three experiments. (E) Binding of the Hsp70 system comprising Ssa1 and the indicated concentrations of the Ydj1 (left panel), or Sis1 (right panel) to the sensor-bound heat-aggregated luciferase. (F) Binding of Hsp70 system comprising Ydj1 (left panel) or Sis1 (right panel) and Ssa1 at the indicated concentrations to the sensor-bound heat-aggregated luciferase. (G), (H), (I), (J) Upper panels show experimental schemes. (G) Binding of Ssa1 with Ydj1 or Sis1 to the sensor bound heat-aggregated GFP. (H) Binding of Ssa1 and Ydj1 or Sis1 to heat-aggregated proteins from the soluble fraction of yeast lysate. (I) Binding of the Hsp70 system comprising Ssa1 WT or the T201A V435F variant and Ydj1 or Sis1 to the sensor-bound heat-aggregated luciferase. (J) Binding of Sis1 WT or Sis1 F201H to the sensor-bound heat-aggregated luciferase. (E)-(J) Dashed lines indicate the start of the incubation and dissociation steps.

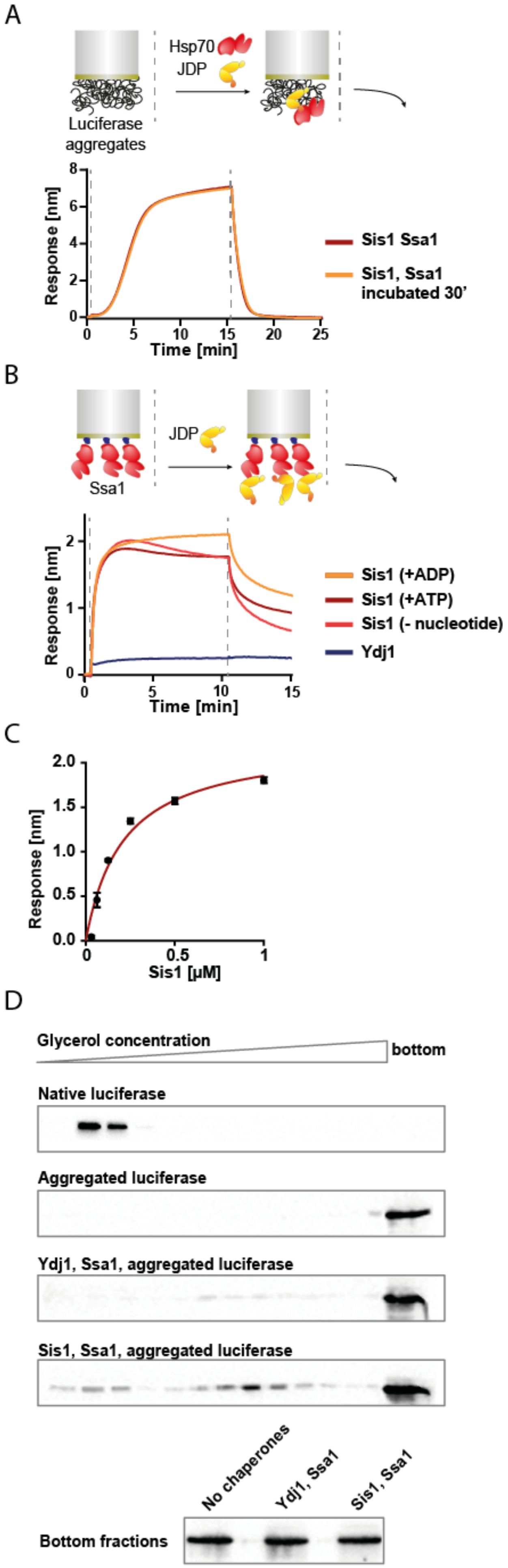

A
Hsp70
JDP
Luciferase aggregates
Response [nm]
Time [min]
Sis1 Ssa1
Sis1, Ssa1 incubated 30'
B
JDP
Ssa1
Response [nm]
Time [min]
Sis1 (+ADP)
Sis1 (+ATP)
Sis1 (- nucleotide)
Ydj1
C
Response [nm]
Sis1 [μM]
D
Glycerol concentration
bottom
Native luciferase
Aggregated luciferase
Ydj1, Ssa1, aggregated luciferase
Sis1, Ssa1, aggregated luciferase
No chaperones
Ydj1, Ssa1
Sis1, Ssa1
Bottom fractions

Supplementary Figure 2. (A), (B) Upper panels show experimental schemes. (A) Initial incubation of Sis1 and Ssa1 does not influence binding to the sensor-bound heat-aggregated luciferase. Sis1 and Ssa1 were incubated for 30 mins prior to the addition to the sensor-bound luciferase aggregates (yellow) or mixed at the start of the reaction (red). (B) Binding of J-domain proteins, as indicated in the legend, to the sensor-bound His_6-SUMO-Ssa1, in the presence or absence of nucleotides, as indicated. (A),(B) Dashed lines indicate the starting point of the incubation and dissociation steps. (C) Apparent dissociation constant determined based on equilibrium levels of Sis1 binding to His_6–SUMO-Ssa1 immobilized on the BLI sensor (K_D^{app} = 210 ± 32 nM, B_{max}^{app} = 2.24 ± 0.13 nm). Sis1 was used at the concentrations: 31.25 nM, 62.5 nM, 125 nM, 250 nM, 500 nM and 1000 nM. Points indicate mean with SD from three independent experiments. The *One site – specific binding*, *$Y=B_{max}*X/(K_D+X)$* model (solid red line) was fitted to the data with *Least squares* approximation using the *GraphPrism* software. (D) Analysis of sedimentation properties of aggregated luciferase. Aggregated or native luciferase was incubated for 1h at 25°C with or without the Hsp70 chaperone system (Ssa1 and Ydj1 or Sis1) and sedimented in glycerol gradient. The fractions were analysed with Western blot using anti-Luciferase antibodies. Lower panel shows the direct comparison of the bottom fractions.

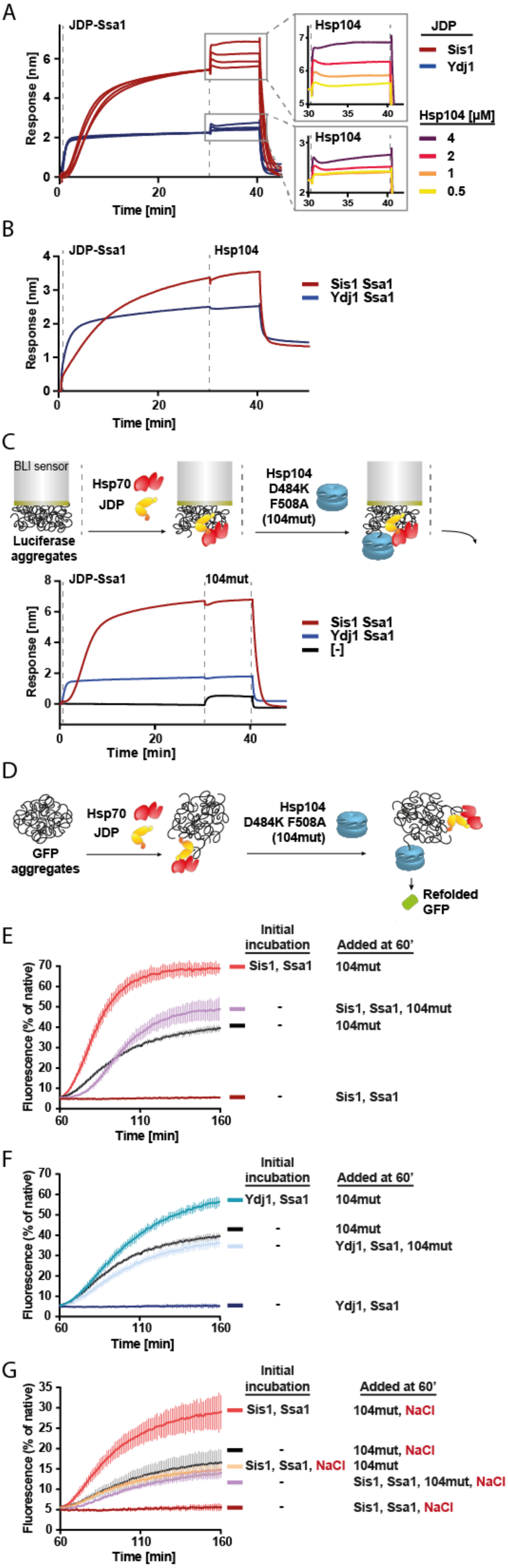
A
JDP-Ssa1
Response [nm]
Time [min]
Hsp104
JDP
Sis1
Ydj1
Hsp104 [μM]
4
2
1
0.5
B
JDP-Ssa1
Hsp104
Response [nm]
Time [min]
Sis1 Ssa1
Ydj1 Ssa1
C
BLI sensor
Luciferase aggregates
Hsp70
JDP
Hsp104 D484K F508A (104mut)
JDP-Ssa1
104mut
Response [nm]
Time [min]
Sis1 Ssa1
Ydj1 Ssa1
[-]
D
GFP aggregates
Hsp70
JDP
Hsp104 D484K F508A (104mut)
Refolded GFP
E
Fluorescence (% of native)
Time [min]
Initial incubation
Added at 60'
Sis1, Ssa1
104mut
-
Sis1, Ssa1, 104mut
-
104mut
-
Sis1, Ssa1
F
Fluorescence (% of native)
Time [min]
Initial incubation
Added at 60'
Ydj1, Ssa1
104mut
-
104mut
-
Ydj1, Ssa1, 104mut
-
Ydj1, Ssa1
G
Fluorescence (% of native)
Time [min]
Initial incubation
Added at 60'
Sis1, Ssa1
104mut, NaCl
-
104mut, NaCl
Sis1, Ssa1, NaCl
104mut
-
Sis1, Ssa1, 104mut, NaCl
-
Sis1, Ssa1, NaCl

Supplementary Figure 3. (A) BLI sensors covered with luciferase aggregates and Sis1-Ssa1 (red curves) and Ydj1-Ssa1 (blue curves) were incubated with Hsp104 at the concentrations indicated in the right inlet panels, showing the enlarged Hsp104-binding step. (B) Hsp104 binding to the crosslinked luciferase aggregates incubated with Ydj1-Ssa1 (blue) or Sis1-Ssa1 (red). (C) Upper panel shows the experimental scheme. Hsp104 D484K F508A binding to the sensor-bound luciferase aggregates in the presence or absence of the Hsp70 system (Ssa1 and Sis1 or Ydj1), as indicated in the legend. (A), (B), (C) Dashed lines indicate the starting point of the incubation and dissociation steps. (D) Experimental scheme for panels E, F, G. (E) The impact of the initial incubation with Ssa1 and Sis1 on the reactivation of heat-aggregated GFP, as indicated in the legend. (F) The effect of the initial incubation with Ssa1 and Ydj1 on the reactivation of heat-aggregated GFP, as indicated in the legend. (G) 120 mM NaCl was added to the reaction mixture to inhibit the Hsp70 system as indicated in red in the legend. The fluorescence signal (panels E, F, G) was normalized to non-aggregated GFP. Error bars show standard deviation from three experiments.

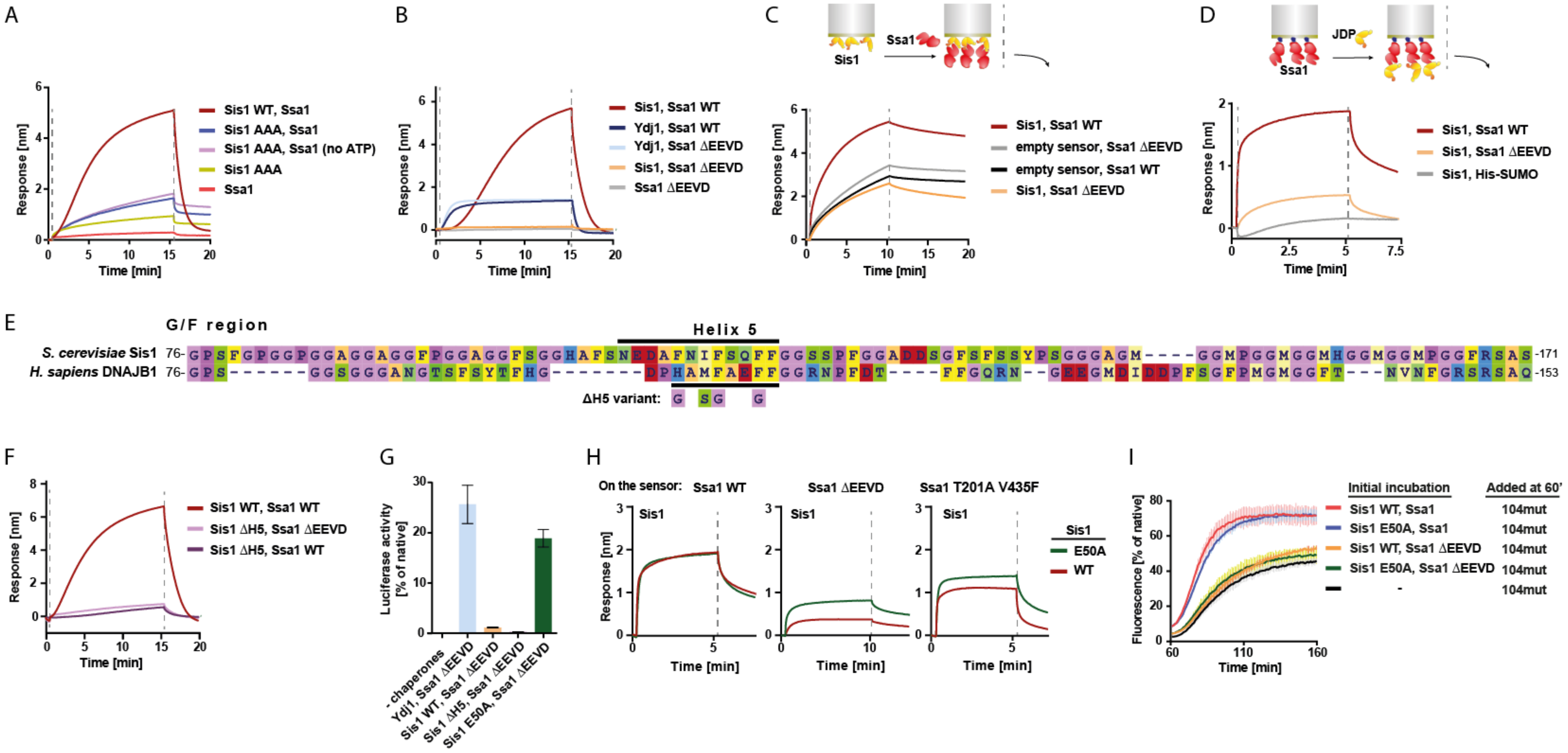
A
Response [nm]
Time [min]
Sis1 WT, Ssa1
Sis1 AAA, Ssa1
Sis1 AAA, Ssa1 (no ATP)
Sis1 AAA
Ssa1
B
Response [nm]
Time [min]
Sis1, Ssa1 WT
Ydj1, Ssa1 WT
Ydj1, Ssa1 ΔEEVD
Sis1, Ssa1 ΔEEVD
Ssa1 ΔEEVD
C
Sis1
Ssa1
Response [nm]
Time [min]
Sis1, Ssa1 WT
empty sensor, Ssa1 ΔEEVD
empty sensor, Ssa1 WT
Sis1, Ssa1 ΔEEVD
D
Ssa1
JDP
Response [nm]
Time [min]
Sis1, Ssa1 WT
Sis1, Ssa1 ΔEEVD
Sis1, His-SUMO
E
G/F region
Helix 5
S. cerevisiae Sis1 76- GPSFGPGGPGGAGGAGGFPGGAGGFSGGHAFSNEDAFNIFSQFFGGSSPFGGADDSGFSFSSYPSGGGAGM----GGMPGGMGGMHGGMGGMPGGFRSAS -171
H. sapiens DNAJB1 76- GPS------GGSGGGANGTSFSYTFHG--------DPHAMFAEFFGGRNPFDT----FFGQRN--GEEGMDIDDPFSGFPMGMGGFT---NVNFGRSRSAQ -153
ΔH5 variant: G SG G
F
Response [nm]
Time [min]
Sis1 WT, Ssa1 WT
Sis1 ΔH5, Ssa1 ΔEEVD
Sis1 ΔH5, Ssa1 WT
G
Luciferase activity [% of native]
- chaperones
Ydj1, Ssa1 ΔEEVD
Sis1 WT, Ssa1 ΔEEVD
Sis1 ΔH5, Ssa1 ΔEEVD
Sis1 E50A, Ssa1 ΔEEVD
H
On the sensor:
Ssa1 WT
Ssa1 ΔEEVD
Ssa1 T201A V435F
Sis1
Response [nm]
Time [min]
Sis1
E50A
WT
I
Fluorescence [% of native]
Time [min]
Initial incubation
Added at 60'
Sis1 WT, Ssa1
Sis1 E50A, Ssa1
Sis1 WT, Ssa1 ΔEEVD
Sis1 E50A, Ssa1 ΔEEVD
-
104mut

Supplementary Figure 4. (A) Binding of Ssa1 alone or with Sis1 WT or the Sis1 AAA (mutation in the HPD motif) variant, as indicated in the legend, to the sensor-bound luciferase aggregates. (B) Binding of the Hsp70 system comprising Ssa1 or Ssa1 ΔEEVD and Sis1 or Ydj1, as indicated, to the sensor-bound luciferase aggregates. (C), (D) Upper panel shows the reaction scheme. (C) Binding of Ssa1 or Ssa1 ΔEEVD variant to the sensor-bound Sis1 or an empty BLI-sensor, as indicated in the legend. (D) Binding of Sis1 to the sensor-bound Ssa1 (red) or Ssa1 ΔEEVD (yellow) or His_6–SUMO (grey). Ssa1 variants were immobilized on the sensor through the His_6-SUMO tag. (E) Alignment of the G/F region of *Saccharomyces cerevisiae* Sis1 and *Homo sapiens* DnaJB1. Amino acid substitutions introduced in the helix 5 in order to generate the ΔH5 variants are indicated below the respective residues. Black lines indicate the length of the helix 5 in each ortholog, according to the AlphaFold structure (Sis1, AF-P25294-F1) (Tunyasuvunakool, Adler et al., 2021) or NMR structure (DNAJB1, 6Z5N) (Faust, Abayev-Avraham et al., 2020)(F) Binding of Ssa1 or Ssa1 ΔEEVD with Sis1 or Sis1 ΔH5 to the sensor-bound luciferase aggregates. (G) Activity of aggregated luciferase after 1 h of incubation with the indicated chaperones. The results presented in the panel represent averages with SD from three independent experiments. (H) Binding of Sis1 and Sis1 E50 sensor-bound Ssa1 WT (left panel), Ssa1 ΔEEVD (middle panel) or Ssa1 T201A V435F (right panel). The Ssa1 variants were immobilized on the BLI sensor through His_6-SUMO tag. (I) The impact of the initial incubation (1 h) with the indicated chaperones on subsequent reactivation of heat-aggregated GFP by the Hsp104 D484K F508A disaggregase. The fluorescence signal was normalized to non-aggregated GFP. Error bars show standard deviation from three experiments. Experimental scheme is presented in the Supplementary Fig. 3D.

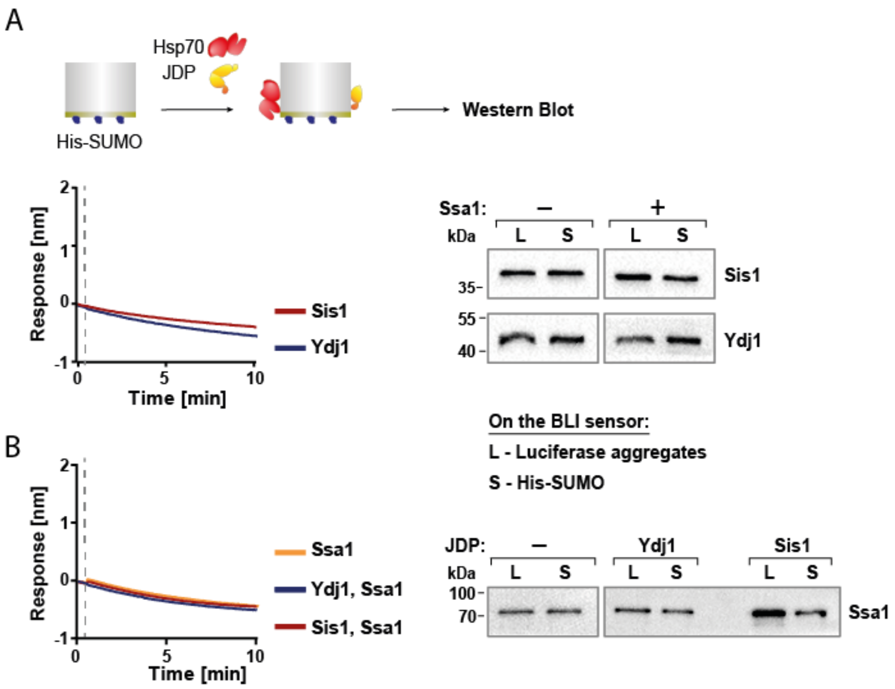

Supplementary Figure 5. Binding of (A) the JDPs alone and (B) the JDPs with Ssa1 or Ssa1 alone to the sensor-bound His_6-SUMO (left panels). After 10 mins, the interaction of chaperones with the sensor-bound His_6-SUMO or luciferase aggregates was assessed with Western Blot using the chaperone-specific antibodies, as indicated in the legend (right panels).

Supplementary methods

Bio-layer interferometry

Binding of chaperones to aggregated yeast proteins. Preparation of cell lysate: Overnight W303 yeast cells cultured in the YPD medium were pelleted and resuspended in the buffer A (25 mM HEPES-KOH pH 8.0; 75 mM KCl; 15 mM $MgCl_2$) with an addition of zymolyase (2 mg per 1 g of the yeast cell pellet). After 30 mins of incubation at 37 °C and lysis with French Press, the lysate was centrifuged at 4 °C for 30 mins at 30 000 rpm. Protein concentration in the supernatant was 10 mg/ml. Sensor preparation: Dip and Read® Ni-NTA biosensor, after initial hydration in the buffer A, was immersed for 10 minutes in the buffer A containing 6M urea and 8.2 µM His-tagged luciferase. Following this time, binding of luciferase reached saturation at ~6 nm of the bio-layer thickness. Next, the sensor was washed with the buffer A for 5 minutes, transferred to the buffer A containing soluble yeast proteins (5 mg/ml) and incubated for 10 minutes at 55 °C. This temperature exceeds the melting point of the major fraction of the yeast proteome (Jarzab, Kurzawa et al., 2020). The resulting aggregate assembly on the sensor was ~30 nm-thick. The biosensor was then equilibrated for 30 minutes in the buffer A containing 2 mM DTT and 5 mM ATP. Chaperone binding and dissociation was performed in the same buffer.

Direct protein-protein interactions. The Dip and Read® Ni-NTA biosensor was hydrated in the buffer A. An initial baseline of 30 s was performed in the buffer A with 2 mM DTT and 5 mM ATP, then the biosensor was immersed for 10 minutes in the buffer A with 2 mM DTT, 5 mM ATP and 0.5 µM His-SUMO-Ssa1, or 0.5 µM His-SUMO-Ssa1 ΔEEVD, or 1 µM His-SUMO-Ssa1 T201A V435F or 0.4 µM His-Sis1 or 0.9 µM His-SUMO, which served as a control. Saturation was reached at ~6 nm for His-SUMO-Ssa1 and its variants, ~12 nm for His-Sis1 and ~3 nm for His-SUMO. Next, the sensor was washed with the buffer A with 2 mM DTT and 5 mM ATP for 2 minutes, and then immersed in the same buffer with the indicated chaperones at 1 µM concentration. The dissociation step was performed in the same buffer.

Heat-aggregated GFP renaturation assay

GFP disaggregation was performed as previously described (Zietkiewicz, Krzewska et al., 2004). All chaperones were used at 1 µM concentration. In the GFP-remodelling experiments (Supplementary Figs 3E, F, G and 4I) chaperones were used at 1 µM, except Hsp104 D484K F508A, which was at 0.15 µM. Fluorescence was measured using the JASCO FP-8000 Fluorescence Spectrometer or Beckman Coulter DTX 880 Plate Reader. The curves presented in the figures represent average with standard deviation from at least three independent experiments.

Analysis of aggregate remodelling with sedimentation

240 µM luciferase was diluted to 30 µM in the buffer A containing 6M urea and incubated at 25 °C for 15 minutes. Next, it was transferred to 48 °C for 10 minutes, rapidly 25-fold diluted with the buffer A and incubated at 25 °C for 15 minutes. Such prepared luciferase aggregates were 4- fold diluted into reaction mixtures. Reaction mixture contained 0.3 µM of aggregated or native luciferase (control) and indicated chaperones at 1 µM concentration in the buffer A containing 2 mM DTT and 5 mM ATP. Reaction mixtures were incubated at 25 °C for 1 h and applied onto 3.5 ml 10-60% (v/v) glycerol gradient in the same buffer. The gradients were sedimented at 4 °C using the Beckman SW60 rotor for 15h at 35 000 rpm. The gradients were fractionated and analysed with Western blot.

Western blot

Chaperone binding to the aggregate-covered sensor was examined as in the BLI experiment, however just before the dissociation step, the sensor was removed from the reaction buffer and incubated in the Laemmli buffer (4% SDS, 20% glycerol, 10% 2-mercaptoethanol, 0.004% bromophenol blue and 0.125 M Tris HCl, pH 6.8) containing 50 mM EDTA for 10 min at 100 °C. SDS-PAGE and immunoblotting was carried out according to the standard procedures.

Chaperone binding to the sensor surface outside the bio-layer (e.g. glass walls), was assessed using sensors with immobilized His_6-SUMO, with which chaperones do not interact, according to the BLI signal. Such unspecific binding was detected with Western Blot for all chaperones (Supplementary Fig. 5A, B). Such strong background limits the sensitivity of the Western Blot analysis of the sensor-bound proteins only to those most abundant, such as Ssa1 in the case of the aggregate-interacting Sis1-Ssa1 system (Supplementary Fig. 5B).

Rabbit anti-sera specific for Ssa1, Ydj and Sis1 were used as primary antibodies. HRP conjugated anti-rabbit IgG (Bio-Rad) were used as secondary antibodies. Blots were developed using SuperSignal West Pico Chemiluminescent Substrate (Thermo Scientific), scanned using ChemiDoc MP Imaging System (Bio-Rad) and quantified using Image-Lab software (Bio-Rad).

References

Faust O, Abayev-Avraham M, Wentink AS, Maurer M, Nillegoda NB, London N, Bukau B, Rosenzweig R (2020) HSP40 proteins use class-specific regulation to drive HSP70 functional diversity. *Nature* 587: 489-+

Jarzab A, Kurzawa N, Hopf T, Moerch M, Zecha J, Leijten N, Bian Y, Musiol E, Maschberger M, Stoehr G, Becher I, Daly C, Samaras P, Mergner J, Spanier B, Angelov A, Werner T, Bantscheff M, Wilhelm M, Klingenspor M et al. (2020) Meltome atlas-thermal proteome stability across the tree of life. *Nat Methods* 17: 495-503

Tunyasuvunakool K, Adler J, Wu Z, Green T, Zielinski M, Žídek A, Bridgland A, Cowie A, Meyer C, Laydon A, Velankar S, Kleywegt GJ, Bateman A, Evans R, Pritzel A, Figurnov M, Ronneberger O, Bates R, Kohl SAA, Potapenko A et al. (2021) Highly accurate protein structure prediction for the human proteome. *Nature* 596: 590-596

Zietkiewicz S, Krzewska J, Liberek K (2004) Successive and synergistic action of the Hsp70 and Hsp100 chaperones in protein disaggregation. *J Biol Chem* 279: 44376-83
